# Digital versus traditional workflows for fabrication of implant-supported rehabilitation: A systematic review

**DOI:** 10.6026/9732063002001075

**Published:** 2024-09-30

**Authors:** Gaurang Mistry, Asha Rathod, Sapna Singh, Ashwini Kini, Kunal Mehta, Rishabh Mistry

**Affiliations:** 1 Department of Prosthodontics, D.Y. Patil Deemed to be University, School of Dentistry, Navi Mumbai, Maharashtra, India

**Keywords:** Dental implants, rehabilitation, digital workflows, computer-aided design

## Abstract

Conventional analog methods were extensively followed for creating implant-supported prostheses. The advent of digital technologies has replaced these methods. This systematic review and meta-analysis aimed to investigate the clinical efficiency and patient acceptance associated with digital and traditional workflows in implant-supported rehabilitation. Multiple electronic databases were searched for studies published between 2010 and mid-2023. The protocol number of the study was PROSPERO CRD CRD42023471411. Two independent reviewers selected studies, evaluated data, and assessed the risk of bias. A fixed effect model was used for meta-analysis, and summary effects were calculated by odds ratio (OR) and 95% CI. The pooled values for included studies in the meta-analysis were as follows: taste (-4.38 [-6.56, -2.20]), anxiety (-0.83 [-1.57, -0.10]), pain (-1.35 [-2.75, 0.05]), and discomfort (-1.28 [-3.23, 0.67]), indicating reduced complaints for these domains with digital methods (p < 0.05). The digital techniques provided better patient satisfaction and time efficiency. Digital workflows in implant-supported rehabilitation showed better patient satisfaction and reduced procedural discomfort, substantiating a paradigm shift towards digital methodologies.

## Background:

Prosthodontics as a clinical specialty has revolutionized with time and so has the procedures for creating implant-supported prostheses. Traditionally, conventional analog methods were extensively followed [1]. The advent of digital technologies has replaced these methods. The methodological shift has given rise to the need to investigate the operational effectiveness and patient acceptance associated with these two workflows. In order to bring about the best patient outcomes in dental practice, it becomes crucial for a thorough evaluation of digital versus traditional workflow. The success of any rehabilitation process depends on patient experience and preferences [2]. Digital workflow provides a novel set of experiences for patients, ranging from the convenience of digital impressions to reducing chairside time. Understanding the concepts of patient acceptance, satisfaction and preferences concerning digital and traditional workflows is crucial for ensuring patient-centric care. The systematic review intended to fill the current knowledge gap by synthesizing and critically examining the available literature on the clinical efficiency and patient preferences linked to digital and traditional workflows for implant-supported rehabilitations. Evidence so obtained will provide a comprehensive understanding of the advantages, disadvantages, and scope for improvement in both digital and traditional approaches. The findings of the present review will facilitate evidence-based decision-making by upgrading the existing knowledge of clinicians about implant-related workflow. The review will also help in understanding the interaction between technology, expected clinical outcomes, and patient satisfaction, thereby laying a strong foundation for the future of implant-supported rehabilitation workflows.

## Materials and Methods:

The present systematic review and meta-analysis were performed according to the guidelines of Preferred Reporting Items for Systematic Review 2020 (PRISMA 2020), (protocol number PROSPERO CRD CRD42023471411). The following focused question in the Patient, Intervention, Comparison, and Outcome (PICO) format was proposed "Is there a difference in the clinical Efficiency and Patient Preferences outcomes for Digital Workflows as compared to Traditional Workflows for fabrication of implant-supported rehabilitation?"

The systematic review included cohort studies, cross-sectional studies, clinical trials, in-vivo studies, randomized clinical trials, controlled clinical trials, non-randomized clinical trials, quasi-experimental studies and non-experimental studies which compared the clinical efficiency and patient preferences outcomes for digital workflows to traditional workflows. Multiple electronic databases were searched for studies published between 2010 and July 2023. Databases searched were Cochrane Central Register of Controlled Trials (CENTRAL), MEDLINE, CINAHL, EMBASE, PsycINFO, Scopus, ERIC and Science Direct with controlled vocabulary and free text terms.

The following search strategies were used. Population -(((Dental Implant [MeSH Terms] OR Dental implant [MeSH Terms] OR dental implants [MeSH Terms] OR Implant [MeSH Terms] OR implants [Text Word] AND dental [Text Word] OR Dental Prosthesis [Text Word] OR Dental prosthesis, crown, dentures [Text Word] OR Implant-supported, superstructure [Text Word] OR fixed [Text Word] OR removable [Text Word] AND reconstruction [Text Word] OR restoration [Text Word])). Intervention-(("Dental technology" [MeSH Terms])) OR ("Computer-aided design" [MeSH Terms])) OR (Digital workflow [Text Word]) OR (virtual [Text Word] OR cad/cam, impression [Text Word] OR intraoral scan [Text Word] OR optical, guided [Text Word] AND planning [Text Word])), Comparison-((Conventional[Text Word] OR analog [Text Word] OR traditional[Text Word])).Outcome-(Success [Text Word] OR Pain [Text Word] OR Burning Sensation [Text Word] OR Mouth opening [Text Word] OR Mouth opening [Text Word] OR Interincisal Distance [Text Word] OR commissural width [Text Word] ). Study Designs-((Visual analog scale [MeSH Terms] OR patient perception [MeSH Terms] OR PROMs [Text Word] OR Patient-centered outcome [Text Word] OR VAS [Text Word])). Combination Term AND was used between the PICOS terms.

**Figure 1:**
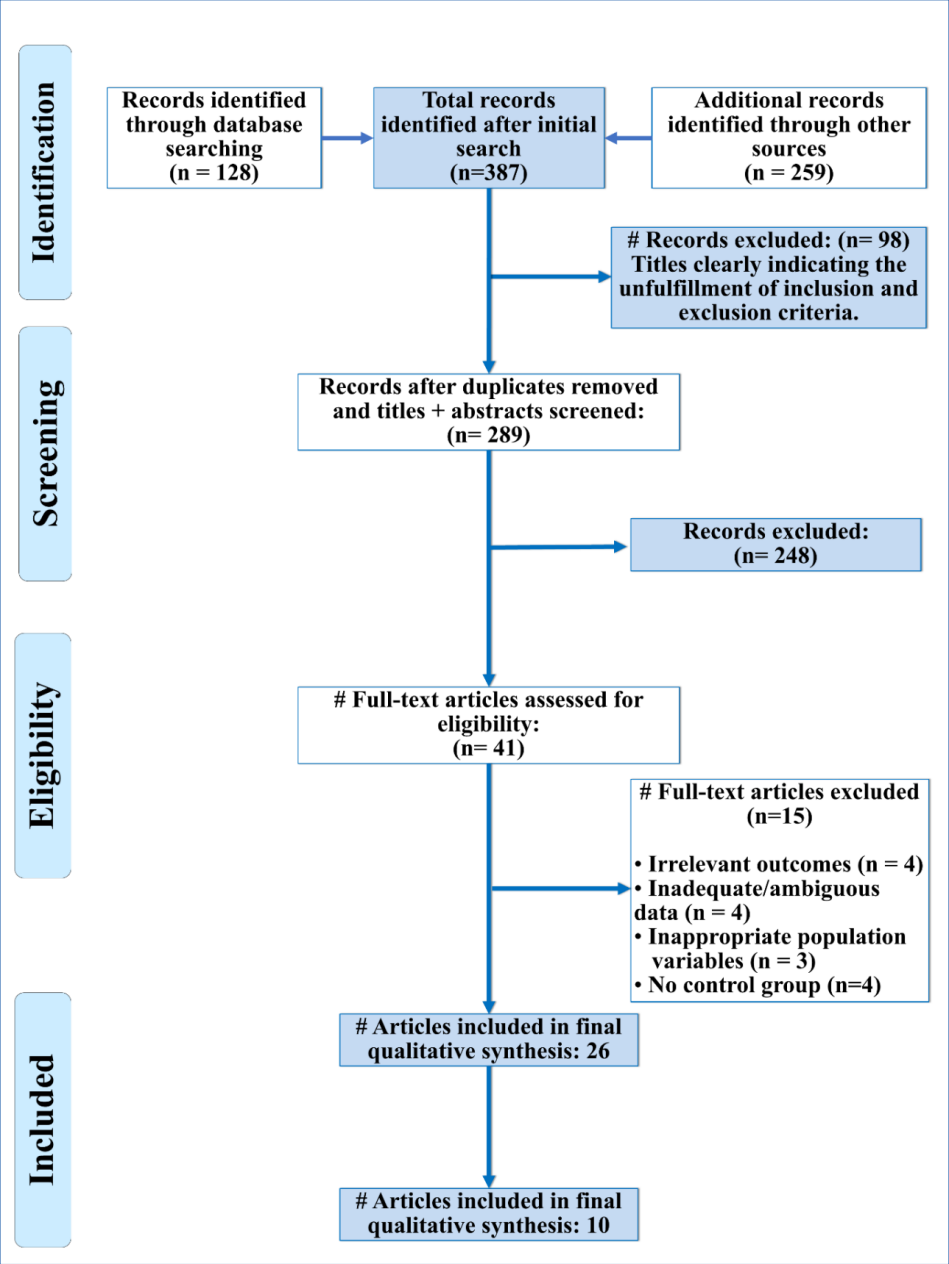
PRISMA Flow Diagram

The initial electronic database resulted in 387 titles, Duplicate records were removed. The level of concordance, calculated through Cohen's kappa, between the two reviewers was 0.90 for titles and abstracts and 0.92 for full texts. Discrepancies among authors/reviewers were resolved by the third author (GM) through careful discussion. Review reports, case series, *in-vitro* and animal studies, single intervention studies without the comparative group, Trials involving participants who had a history of significant medical conditions, or under any medication that could have influenced study results, trials involving a combination of treatment other than digital workflow in the intervention group were excluded. After 108 duplicate references were removed, 279 abstracts were screened and 58 relevant titles were selected by two independent reviewers. Following examination and discussion by the reviewers, 21 articles were selected for full-text evaluation. Hand-searching of the reference lists of the selected studies did not deliver additional papers. After pre-screening, application of the inclusion and exclusion criteria, and handling of the PICO questions, 10 studies were included in the qualitative synthesis and 7 studies were included for quantitative assessment (Figure 1). Studies published in any language where the English translation is possible and studies with full-text articles were included.

## Data extraction:

Two reviewers independently extracted data from the included studies. Disagreements were again resolved through discussion. Data gathered was carried out using a list of items. These included authors, year and title of study, country, study design, sample size, age group of participants, gender (Table 1) Details regarding the publication, the participants, settings, interventions, comparators, outcome measures, study design, statistical analysis, results and all other relevant data were carefully and accurately extracted from all included studies.

## Methodological quality assessment:

**Figure 2:**
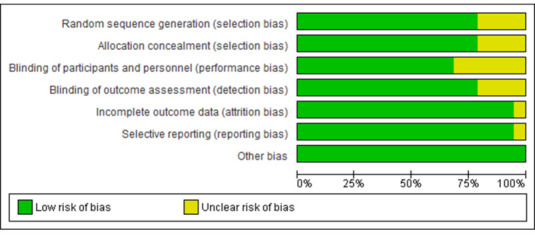
Risk of bias graph

**Figure 3:**
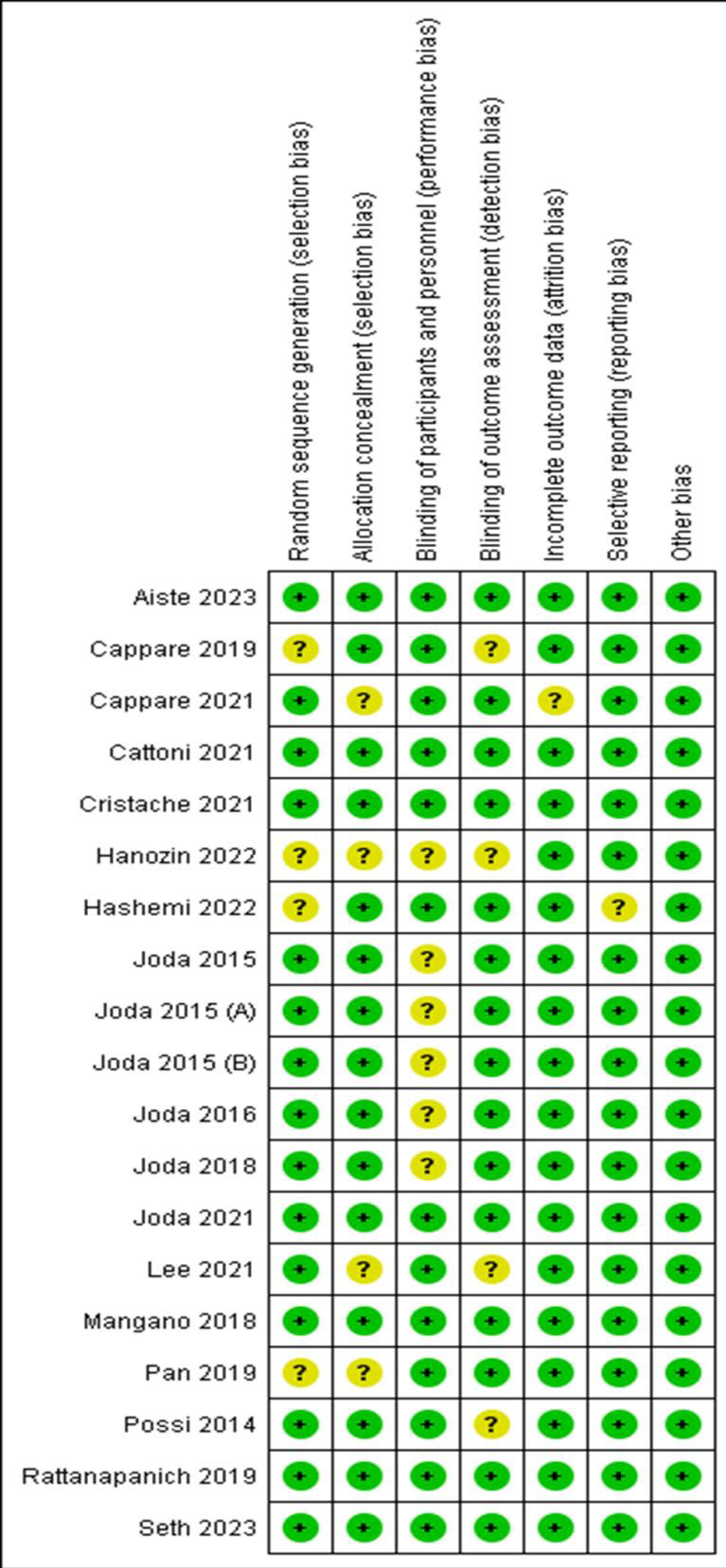
Risk of bias summary

For randomized controlled trials, Cochrane RoB-2 tool 2 was used for quality assessment. According to this tool, the risk of bias was assessed at the study level under seven domains: random sequence generation, allocation concealment, blinding of participants and personnel, blinding of outcome assessment, incomplete outcome data, selective reporting, and other biases. The overall risk for individual studies was assessed as low, moderate, or high risk based on domains and criteria. The study was assessed to have a low overall risk only if all domains were found to have low risk. High overall risk was assessed if one or more of the six domains were found to be at high risk. A moderate risk assessment was provided to studies when one or more domains were found to be uncertain, with none at high risk (Figure 2 and Figure 3).

The risk of bias was evaluated using RevMan (Review Manager Version 5.3) software. Quality assessment of non-randomized studies was done using the Methodological Index for Non-Randomized Studies (MINORS) tool [3]. This includes an eight-item assessment for noncomparative-randomized studies. The items were scored 0 (not reported), 1 (reported but inadequate), or 2 (reported and adequate). The global ideal score is 16 for non-comparative studies and 24 for comparative studies (Table 2: Quality assessment according to MINORS tool). Among the included RCTs, fifteen studies showed low risk, three studies showed moderate risk and one study showed high risk of bias. In a study by Hanozin 2022, information about randomization, allocation concealment and blinding of participants and personnel was unclear leading to a high risk of bias.

## Data synthesis:

Data synthesis was carried out using a descriptive synthesis, with a summary of the characteristics of each included study. For quantitative synthesis, a summary of the combined estimate related to the intervention effect was calculated as a mean of the differences in the effects of post-intervention in individual studies.

Review Manager (RevMan) 5.3 used statistical analysis for quantitative synthesis. The combined results were expressed as mean and standard deviation for the continuous data at 95% confidence intervals (CIs) and p<0.05 was considered significant. Tau-square and I^2^ test was used to assess the heterogenicity of the included studies. Assessment of clinical heterogeneity refers to differences between studies about the participants, interventions, comparators, settings and outcomes. For I^2^>50%, the random-effects model was applied. Subgroup analysis was performed to reduce the sources of clinical heterogeneity among the studies. Also, the statistical significance was set at p-value (two-tailed) <0.05. Standardized mean difference (SMD) was used as an effect measure as the studies had to assess the same outcome but measure it in a variety of ways. The studies featured different `study characteristics like taste, anxiety, nausea, pain, discomfort and overall patient satisfaction. It also featured time efficiency and marginal bone loss. Meta-analysis was conducted only for those studies featuring variables that could be grouped. Data was extracted for the categorical variable of different workforces (Digital vs conventional). For other studies, a narrative synthesis of the data was conducted. Publication bias was not quantitatively evaluated by the Egger test or funnel plot, as there were not enough studies to be grouped in a funnel plot.

## Results:

## Study characteristics:

Twenty-six studies [4, 5, 6 7, 8, 9, 10, 11, 12, 13, 14, 15, 16, 17, 18, 19, 20, 21, 22, 23, 24, 25, 26, 27, 28-29] were included in this systematic review. These studies were conducted in different parts of the world with Turkey, Italy, Switzerland, Korea, Belgium, China, Thailand, Rome, USA, Romania, Boston, Iran, Zurich, and Denmark. Among the included studies, n=19 were RCTs and n=7 were non-RCTs. Different types of digital techniques were used in these studies such as IOS plus CAD/CAM technology, TRIOS Pod system, CEREC AC Omnicam, Carestream 3600, 3-Shape, i-Tero Element, etc. For the conventional technique, the impression was made using polyether impression or gypsum cast or alginate material. The conclusions of all studies indicated that digital techniques provide more patient satisfaction as compared to conventional techniques. The digital techniques are also time efficient.

## Quality assessment of RCTs:

Among the included RCTs, fifteen studies showed low risk, three studies showed moderate risk and one study showed high risk of bias. In study by Hanozin 2022, information pertaining to randomization, allocation concealment and blinding of participants and personnel was unclear leading to high risk of bias in this study.

## Meta-analysis:

**Figure 4:**
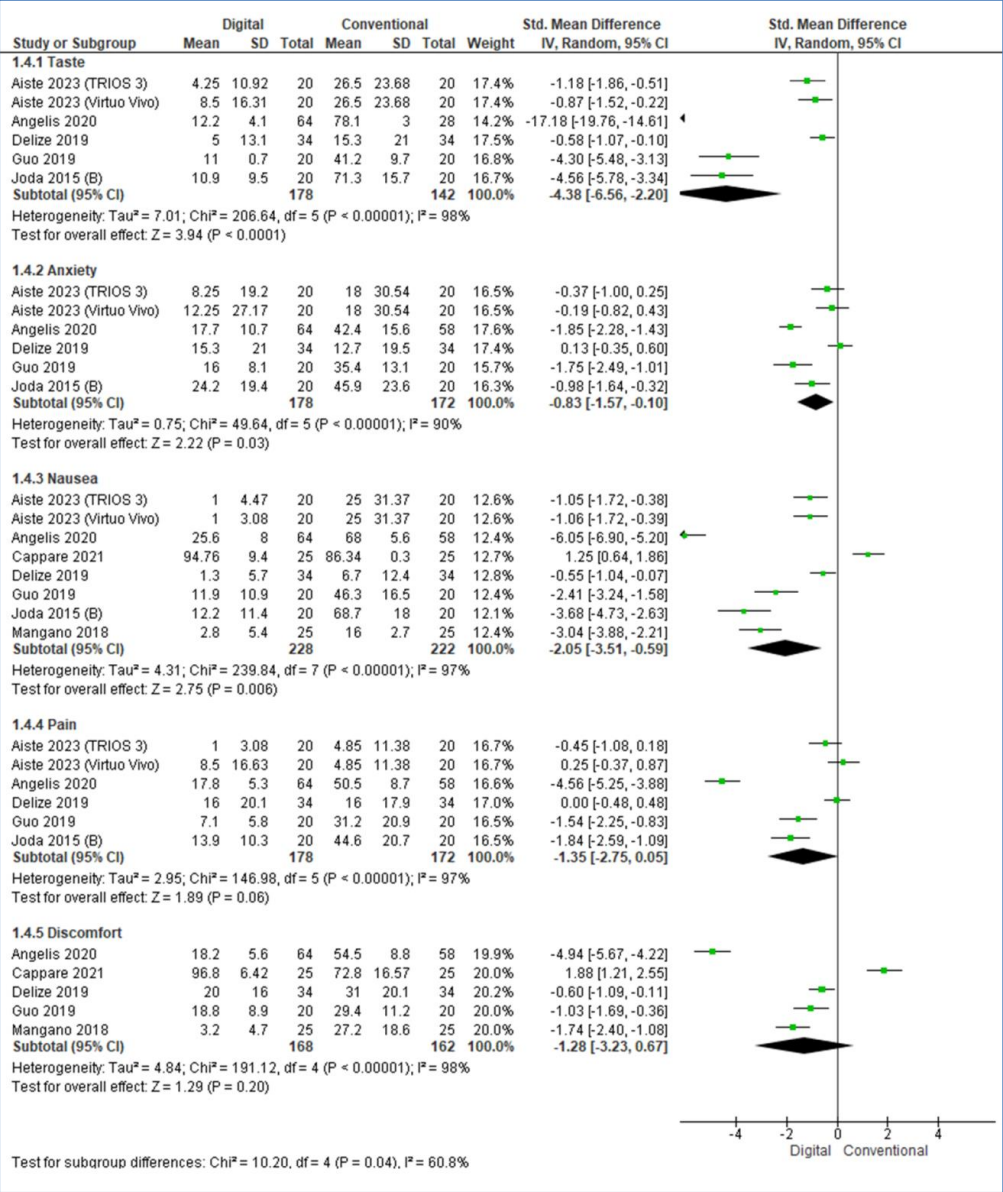
Pooled values for patient-reported outcome measures

Patient-reported outcome measures (Figure 4):

## Taste:

Five studies evaluated taste perception with respect to digital and conventional techniques. The pooled value obtained was -4.38[-6.56, -2.20] indicating that less taste complaints were reported with digital method as compared to conventional. Overall the results were statistically significant (p<0.05) with high heterogeneity (I2=98%).

## Anxiety:

Five studies evaluated anxiety with respect to digital and conventional techniques. The pooled value obtained was -0.83[-1.57, -0.10] indicating that low levels of anxiety were reported with digital method as compared to conventional. Overall, the results were statistically significant (p<0.05) with high heterogeneity (I2=90%).

## Nausea:

Seven studies evaluated nausea with respect to digital and conventional techniques. The pooled value obtained was -2.05[-3.51, -0.59] indicating that low nausea was reported with digital method as compared to conventional. Overall the results were statistically significant (p<0.05) with high heterogeneity (I2=97%).

## Pain:

Five studies evaluated pain with respect to digital and conventional techniques. The pooled value obtained was -1.35[-2.75, 0.05] indicating that less pain complaints were reported with digital method as compared to conventional. Overall the results were statistically significant (p<0.05) with high heterogeneity (I2=97%).

## Discomfort:

Four studies evaluated discomfort with respect to digital and conventional techniques. The pooled value obtained was -1.28[- 3.23, 0.67] indicating that less discomfort was reported with digital method as compared to conventional. Overall the results were not statistically significant (p>0.05) with high heterogeneity (I2=98%).

## Time efficiency:

**Figure 5:**
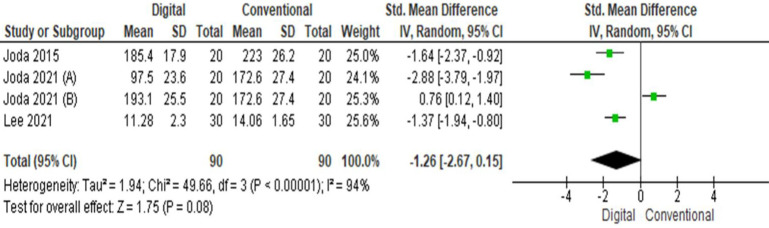
Pooled values for Time efficiency

Four studies evaluated time efficiency with respect to digital and conventional techniques. A total of 90 participants were evaluated in both groups. The pooled value obtained was -1.26[-2.67, 0.15] indicating that overall time required was less with digital method as compared to conventional. Overall, the results were statistically significant (p<0.05) with high heterogeneity (I2=94%) (Figure 5).

## Marginal bone level:

**Figure 6:**
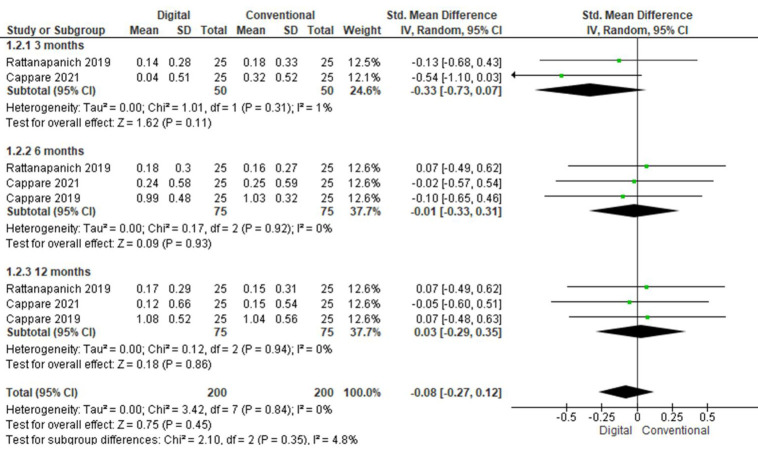
Pooled values for marginal bone levels

Bone level was evaluated at follow-ups of 3, 6 and 12 months (Figure 6).

At three months, two studies were evaluated. The pooled value obtained was -0.33[-0.72, 0.07] indicating that marginal bone levels were less with digital method as compared to conventional at 3 months. Overall the results were not statistically significant (p>0.05) with low heterogeneity (I2=1%). Fixed effect model was used for analysis. At six months, three studies were included. The pooled value obtained was -0.01[-0.33, 0.31] indicating that marginal bone levels were less with digital method as compared to conventional at 6 months. Overall the results were not statistically significant (p>0.05) with low heterogeneity (I2=0%). Fixed effect model was used for analysis. At 12 months, three studies were included. The pooled value obtained was 0.03[-0.29, 0.12] indicating that marginal bone levels were greater with digital method as compared to conventional at 12 months. Overall, the results were not statistically significant (p>0.05) with low heterogeneity (I2=0%). Fixed effect model was used for analysis.

## Overall patient satisfaction:

**Figure 7:**
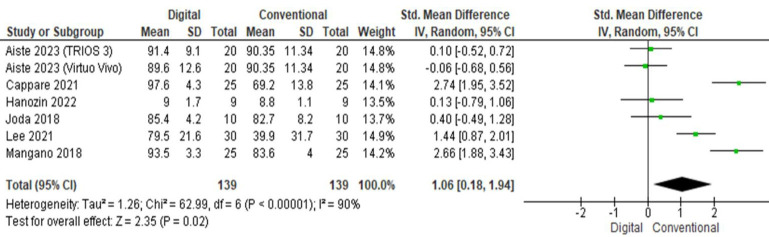
Pooled values for patient satisfaction

Five studies analyzed the overall patient satisfaction regarding digital and conventional methods (Figure 7). The pooled value obtained was 1.06[0.18, 1.94] indicating that the overall satisfaction was greater with digital method as compared to conventional. Overall, the results were statistically significant (p<0.05) with high heterogeneity (I2=90%).

## Discussion:

The current systematic review included a diverse range of randomized as well as non-randomized controlled trials conducted across the globe, reflecting an international perspective on the clinical efficiency and patient preferences related to digital versus traditional workflows for the fabrication of implant-supported rehabilitation. This choice of fabrication materials shows the conventional methods that have been employed for decades [10-13]. The inclusion of both traditional and newer methods allows for a comprehensive comparison, A variety of digital software is employed in the included studies representing digital dentistry. Each technique has its advantages and drawbacks, contributing to the complexity of the digital workflow. The digital workflow, characterized by optical impressions using IOS, digital designing and computer-aided manufacturing of final prostheses, is a recent innovation in contemporary implant treatment [14]. This approach is particularly useful for single crowns and short-span fixed dental prostheses. Both patients and operators have benefitted from the digital workforce. A homogeneous study population was maintained by excluding patients with relevant medical histories and medications, those involving combination treatments that might dilute treatment effects other than the intended treatment thereby controlling for confounders [15-17]. This ensured that the observed differences attributed to the specific methodology employed, thereby increasing the internal validity of our systematic review. Patient-reported outcome measures analyzed in the studies, including taste, nausea, anxiety, and discomfort are indicators of patient experience regarding implant-supported rehabilitation [18,19]. The reliability and validity conclusions supported digital techniques for patient satisfaction. The digital techniques are also time-efficient thereby improving efficiency [15, 25]. Limitations included a lack of comprehensive understanding of the effects of implant therapy from both the patient and operator perspectives. This gap emphasizes the need for a more holistic assessment that considers not only the final functional outcomes but also the entire treatment process and the preferences of both patients and operators. The overall findings revealed evidence favoring the digital approach across various domains. The statistically significant reduction in taste complaints decreased anxiety levels, supported digital processes, it reduced treatment duration, and increased predictability, thereby emphasizing the psychological benefits of digital methodologies [20]. Reduced incidence of nausea and pain, reduction in tissue trauma and postoperative discomfort reported in the digital group shows the enhanced precision and efficiency of digital workflows [21]. However, it is noteworthy that the results were not statistically significant, indicating a need for further research with larger sample sizes and standardized measures to validate these findings. In the periodic evaluation of marginal bone levels, The absence of any significant differences at the three and six-month follow-ups suggests comparable short-term effects of digital and conventional workflows on marginal bone levels Long-term effects of various factors influencing bone levels necessitate continued investigation to draw reliable conclusions.

## Conclusion:

The present systematic review provides a comprehensive overview of the clinical efficiency and patient preferences associated with digital versus conventional workflows in implant-supported rehabilitation. The incorporation of patient-reported outcome measures comprising taste, anxiety, nausea, pain and discomfort, highlighted the multidimensional advantages of digital approaches. The digital techniques provided better patient satisfaction and time efficiency in terms of reduced taste complaints, anxiety levels and procedural discomfort substantiating the paradigm shift towards digital methodologies. The conclusion emphasizes the digital revolution in implant-supported rehabilitation aiming for enhanced clinical efficiency and patient satisfaction. Our findings lay the foundation for further exploration to refine clinical protocols, in patient-centered care transforming digital dentistry.

## Figures and Tables

**Table 1:** Characteristics of included studies

**Study ID**	**Place of study**	**Study design**	**Sample size**	**Age**	**Gender M/F**	**IG**	**CG**	**Outcomes assessed**
Arisan 2010	Turkey	prospective	52 21/16/15	28-63	25/27	bone supported guide (BSG), Stereolithographic guides (SLA)	Standard technique	surgical duration, post-operative pain, swelling, trismus, hemorrhage
Possi 2014	Italy	RCT	51 25/26	28-84	29/22	Implant positioning using the planning software according to anatomic and prosthetic requirements	Conventional technique	treatment time, patient satisfaction, bone loss
Joda 2015	Switzerland	RCT crossover	20/20	34.7-72.8	52.6%/47.4%	Digital workflow using IOS plus CAD/CAM technology	Plaster cast impression technique	time efficiency, number of appointments
Joda 2015 A	Switzerland	RCT crossover	20/20	34.7-72.8	52.6%/47.4%	Digital workflow using IOS plus CAD/CAM technology	Plaster cast impression technique	adjustment time
Joda 2015 B	Switzerland	RCT crossover	20/20	34.7-72.8	52.6%/47.4%	Digital workflow using IOS plus CAD/CAM technology	Plaster cast impression technique	PROMS
Joda 2016 A	Switzerland	RCT crossover	100	19-65	54%/46%	Quadrant-like IOS was taken capturing the 3D implant position and at least two teeth mesially and distally with the TRIOS Pod system	An open-tray approach was used with pre-fabricated stock trays, elastomeric material	time efficiency, operator evaluation
Joda 2018	Switzerland	RCT	20 10/10	mean 55.4 years	25%/75%	A complete digital CAD/CAM-workflow	Fabricated in a combined analog-digital process with individualized	PROMs, FIPS
Mangano 2018	Italy	RCT	50 25/25	24-76	22/28	Optical impression with an intraoral scanner	Conventional impression of the implant with polyvinyl siloxane	peri-implant marginal bone loss, PROMs
Muhlemann 2018	Korea	comparative study	5	24-68	N/A	Each implant was scanned with three different intraoral scan-ners: iTero Cadent (ITE), Lava True Definition (LTD) and Trios 3Shape (TRI)	Conventional gypsum model	precision
Cappare 2019	Italy	RCT	50 patients, 25/25 300 implants	48-72	N/A	Digital scanner was utilized to fabricate the definitive prostheses	Impression material used was gypsum	implant stability, success, peri implant bone loss
Delize 2019	Belgium	non RCT	34	47.5+-1.04	10/21	Digital impressions were performed using IOS (TRIOS® second generation, 3Shape)	Conventional impressions were taken using a closed-tray transfer coping and a heavy- and light-viscosity silicone	PROM, WES index,
Guo 2019	China	prospective clinical study	20	mean 41.4 years	45%/55%	IOS digital impression	conventional implant impression	patient satisfaction, mean time of impression
Pan 2019	China	RCT double blind	40 40/40	mean 45.1	19/21	Digital impression data were digitally transferred to the computer-aided design (CAD) software (3Shape Designer, 3Shape A/S).	a conventional closed-tray implant impression was taken using an implant transfer post and a polyether material	clinical time,
Rattanapanich 2019		RCT	50	49.16+-11.07	12/38	The impressions were recorded while using an intraoral scanner and the data were employed in the computer-assisted design	Conventional technique	implant success, patient satisfaction, marginal bone level
Angelis 2020	Rome	retrospective clinical study	122 64/58	58.3+-6.9	41/81	Digital impression using CEREC AC Omnicam	Conventional impression with alginate impression material	pain, workflow duration, number of appointments, time-efficiency
Chochlidakis 2020	USA	prospective clinical study	16	N/A	N/A	Full-arch intraoral digital scan was obtained with an intraoral scanner	impressions were made using heavy and light body viny polylsiloxane (VPS) material	accuracy of digital impression
Cappare 2021	Italy	RCT	50 25/25	23-65	19/31	impressions were recorded using the CAD/CAM chairside system	temporary prefabricated acrylic resin crowns were obtained and then adapted with an auto-polymerizing acrylic resin	patient satisfaction, plaque index, probing depth, marginal bone levels
Cattoni 2021	Italy	RCT	50 25/25	46-85	N/A	intraoral scanner MyRay matched with CAD software	conventional technique	implant failure, marginal bone level,
Cristache 2021	Romania	RCT	49 24/25 implants 56/55	54.45+-11.11	17/32	Using Carestream 3600 (Carestream Dental LLC, Atlanta, GA, USA) intraoral surface scanner and a digital tooth setup was performed in the CARES software.	with condensation-cured polymethyl siloxane impression material	accuracy of implant insertion, patient feedback, bone loss
Joda 2021	Switzerland	RCT double blind crossover	20 20/20/20	30-76	45%/55%	1. digital workflow using 3-Shape 2. digital workflow using Dental Wings Inc.	conventional workflow using Polyether Impression / Gypsum Cast / Lab-Scan + Exocad Lab Software	time efficiency, cost of treatment
Lee 2021	Boston	RCT crossover	30/30			digital scanning technique was performed by using an IOS (iTero Element; Align Technology Inc)	closed tray impression was made by using an impression coping and polyvinyl siloxane	total time required, accuracy
Hanozin 2022	Belgium	RCT	18 9/9	47.67/57.11	5/13	digital impression (TRIOS®, 3Shape, Denmark	conventional alginate impressions	accuracy of implant position, WES, PES, PROMS
Hashemi 2022	Iran	RCT crossover	10 10/10	47.1+-11	3/7	digital impression (or intraoral scan) of the entire arch was performed with an IoS	open-tray impression technique using one-step putty-light body addition silicone	occlusion, estheic parameters, fabrication time
Aiste 2023	Zurich	RCT double blind	20	30-76	45%/55%	1. digital workflow using 3-Shape 2. digital workflow using Dental Wings Inc.	Mixed analog-digital workflow using polyether /gypsum material	patient satisfaction, dentist evaluation
Pera 2023	Italy	clinical study	9	44-87	5/4	digital impression using a new IOS	traditional impression using impression plaster	Sheffield test to assess passive fitting
Seth 2023	Denmark	RCT crossover	40/40	33-78	22/18	The IOS (CEREC Omnicam; Dentsply Sirona)	polyether impression material (Impregum; 3M ESPE) was used	quality of life, copenhagen index score

**Table 2:** Quality assessment according to MINORS tool

**Study Id**	**A clearly stated aim**	**Inclusion of consecutive patients**	**Prospective collection of data**	**Endpoints appropriate to the aim of the study**	**Unbiased assessment of the study endpoint**	**Follow-up period appropriate to the aim of the study**	**Loss to follow up less than 5%**	**Prospective calculation of the study size**	*An adequate control group	*Contemporary groups	*Baseline equivalence of groups	*Adequate statistical analyses	**Total**
Arisan 2010	2	2	0	1	2	1	2	0	2	2	2	2	18
Muhlemann 2018	2	1	2	2	1	2	2	0	2	2	2	2	20
Delize 2019	2	2	2	1	2	2	2	0	2	2	2	2	21
Guo 2019	2	2	2	2	2	2	2	0	2	2	2	2	22
Angelis 2020	1	2	0	2	2	2	2	2	2	2	2	2	21
Chochlidakis 2020	2	2	2	2	2	2	2	0	2	2	2	2	22
Pera 2023	2	2	1	1	2	2	2	0	2	2	2	2	20
